# Phosphoethanolamine-modified lipid A enables antimicrobial resistance and atypical signaling properties in the marine pathogen *P. damselae* subsp. *damselae*

**DOI:** 10.1128/spectrum.00662-26

**Published:** 2026-07-14

**Authors:** Matthew E. Sherman, Hyojik Yang, David A. Rasko, Joshua Kolb, Anuj Joshi, John Udara Mendis, Helen Dooley, Alison J. Scott, David R. Goodlett, Robert K. Ernst

**Affiliations:** 1 Department of Microbial Pathogenesis, University of Maryland-Baltimore 12265 , Baltimore, Maryland, USA; 2 Department of Biochemistry and Microbiology, University of Victoria, Victoria, British Columbia, Canada; 3 Department of Bioengineering, University of Maryland-College Park 1068, College Park, Maryland, USA; 4 Department of Microbiology and Immunology, Institute of Marine and Environmental Technology, University of Maryland School of Medicine 12264, Baltimore, Maryland, USA; Zhejiang University School of Medicine Sir Run Run Shaw Hospital, Hangzhou, Zhejiang, China; Universidade de Santiago de Compostela-Campus Vida, Santiago de Compostela, Galicia, Spain; Imperial College London, London, United Kingdom

**Keywords:** *Photobacterium*, LPS, lipid A, TLR4, phosphoethanolamine

## Abstract

Lipid A, the immunostimulatory component of lipopolysaccharide (LPS), plays a multitude of roles in host-pathogen interactions. It functions as both a key antigen recognized by innate immune receptors and an essential structural element of the gram-negative bacterial outer membrane. Consequently, gram-negative bacteria exposed to stressful environments can modify their lipid A structure to preserve membrane integrity, evade immune cell recognition, and avoid disruption by antimicrobial agents. In this study, we isolated a strain (SF21) from a nurse shark stool, which was identified as the gram-negative marine pathogen *Photobacterium damselae* subsp. *damselae* via genomic sequencing. Mass spectrometric analysis revealed that its lipid A is extensively modified with phosphoethanolamine, which enabled inherent resistance to the cationic antimicrobial peptide colistin (polymyxin E). Additionally, growth under cold conditions (10°C) led to the replacement of the normally saturated C14 acyl chain in SF21 lipid A with an unsaturated C16:1 acyl chain. Stimulation of NF-κB reporter cells with purified SF21 LPS elicited stronger signaling through human TLR4/MD-2 compared to murine TLR4/MD-2, challenging the paradigm that hexa-acylated LPS is more stimulatory through the murine ortholog of this receptor complex. Altogether, this study characterizes a gram-negative bacterium whose ability to grow in various environments and be detected by the TLR4/MD-2 complex appears linked to its capacity to modify its lipid A.

**IMPORTANCE:**

Gram-negative bacteria can remodel their membrane lipids to accommodate the various stresses that they encounter in the environment. One membrane lipid, called lipid A, is a key structural element of the outer leaflet of their outer membrane. While it provides structural integrity, it also serves as a molecular pattern that our innate immune system utilizes to recognize bacteria and mount an immune response. In this study, we show that a marine pathogen, present in the stool of nurse sharks, possesses lipid A that is modified with phosphoethanolamine. This lipid A modification enabled resistance to antimicrobials, promoted growth at temperatures beyond normal shark body temperature, and induced an altered immune response that differs from prototypical LPS. From this study, we conclude that phosphoethanolamine-modified lipid A improves bacterial fitness, enabling better survival across the various environments that a bacterium may encounter.

## INTRODUCTION

Lipopolysaccharide (LPS), commonly referred to as endotoxin, is an essential component of the gram-negative bacterial outer membrane and serves as a pathogen-associated molecular pattern (PAMP) sensed by eukaryotic innate immune cells (1, 2). LPS is composed of three regions: membrane-bound lipid A, the core oligosaccharide, and the O-antigen (3). During a gram-negative infection, LPS is delivered via accessory proteins to the TLR4/MD-2 receptor complex present on the surface of innate immune cells (4). The lipid A region of LPS mediates binding to TLR4/MD-2 and subsequently results in the dimerization of LPS-bound TLR4/MD-2 complexes, which initiates downstream signaling (5). The NF-κB pathway is primarily activated, which drives the production of pro-inflammatory cytokines, thereby initiating the host innate immune response (6).

Beyond its role as a PAMP, LPS serves as a barrier between the extracellular milieu and bacterial periplasm, making it a target for antimicrobials that perturb outer membrane integrity (7). Lipid A, which anchors LPS to the outer membrane, is amphipathic, containing a disaccharide flanked by negatively charged phosphates attached to nonpolar acyl chains (7). The terminal phosphate moieties are a primary target for cationic antimicrobial peptides (CAMPs), which electrostatically interact with the negatively charged lipid A interface (8). Consequently, gram-negative bacteria utilize inducible LPS modification systems that reduce the overall negative charge of the membrane through the addition of positively charged moieties (9). These modifications include the addition of aminoarabinose or phosphoethanolamine (pEtN). The addition of pEtN onto lipid A is mediated by *eptA* (also known as *pmrC*), which can be activated by the two-component regulatory system PmrAB to express a lipid A pEtN-transferase (7, 9). An alternative mechanism of pEtN addition is via the acquisition of *mcr* (mobile colistin resistance)-encoding plasmids, which express lipid A pEtN-transferases (8).

This ability of gram-negative bacteria to remodel the lipid A region is a key virulence strategy used by bacteria to enhance survival within the host. Specifically, alterations in the acylation state of lipid A modulate TLR4/MD-2–dependent NF-κB activation (6), where hexa-acylated lipid A acts as a potent agonist of TLR4/MD-2 and tetra-acylated lipid A as an antagonist (7, 10). Modification of lipid A acylation functions beyond immune evasion too, in some cases allowing bacteria to adapt to membrane stress through changes in acyl chain composition (9). For example, during cold shock, expression of the lipid A modifying enzyme LpxP is increased, resulting in the replacement of a saturated fatty acid with an unsaturated one, which enhances membrane fluidity during growth in low-temperature conditions (11). Furthermore, orthologs of the TLR4/MD-2 receptor have differential responses to lipid A (5). For instance, the prototypical LPS from *Escherichia coli* (whose lipid A is hexa-acylated) signals more robustly through mouse TLR4/MD-2 compared to human TLR4/MD-2 (10). Altogether, lipid A modifications are at the interface of the host-pathogen interaction, often enabling gram-negative bacteria to better accommodate the varying conditions they may encounter within a host.

In this study, nurse shark stool was cultured at 37°C, yielding a single isolate identified as *Photobacterium damselae* subsp. *damselae* (*Pdd*). LPS analysis of this isolate, designated SF21, revealed a lipid A structure modified with pEtN at the 1-position. Although this modification arose in the absence of CAMP exposure, it conferred intrinsic resistance to colistin, a CAMP that targets lipid A. Additionally, growth under low-temperature conditions induced a structural shift in SF21 lipid A, characterized by replacement of a C14 acyl chain with an unsaturated C16:1 chain. Unlike prototypical diphosphorylated hexa-acylated LPS from *E. coli*, the pEtN-modified LPS from SF21 signaled more robustly through human TLR4/MD-2 as compared to mouse TLR4/MD-2. Altogether, this study highlights the intrinsic antimicrobial resistance conferred by pEtN-modified lipid A and that this modification may alter the established model of sensing of LPS via the mouse versus human TLR4/MD-2.

## MATERIALS AND METHODS

### Reagents

For plate growth, 1.5% Bacto-agar was added to all media. ITO glass slides were obtained from Delta Technologies Ltd. Colistin sulfate salt was obtained from Sigma-Aldrich.

### Bacterial strains, growth conditions, and antimicrobial assay

This *Pdd* strain was isolated from the stool of a nurse shark (*Ginglymostoma cirratum*) obtained postmortem via the cloaca. The female shark, aged between 2 and 3 years and weighing roughly 1 kg, was obtained from Florida coastal waters under a Special Activity License from the Florida Fish and Wildlife Conservation Commission. The animal was flown to Baltimore, where it was maintained for 4 weeks in a 12,000 L tank, containing continuously recirculating seawater at 28°C, within the Institute of Marine and Environmental Technology (IMET), prior to sacrifice. All animal procedures were conducted in accordance with the University of Maryland Baltimore, Institutional Animal Care and Use Committee (IACUC) approved protocol #AUP00000713. A stool sample from the nurse shark was plated on marine agar and incubated at 37°C until colonies were observed. These colonies were then streaked for isolation. Single colonies were then grown in filtered marine broth and stored using standard glycerol stock methods. An individual isolate was sequenced at the Microbial Genome Sequencing Center (SeqCenter) using standard methods (see below) and used for all the experiments in this study. Minimum inhibitory concentration (MIC) analysis was performed as described (12) using a Cerillo heated incubator automatic plate reader. Additionally, *E. coli* BORT (O18ac:K1:H7) was purchased from ATCC and grown at 37°C in lysogeny broth (LB) supplemented with 1 mM MgCl_2_ prior to LPS purification.

### Genome sequencing

This isolate was grown for DNA isolation in marine broth overnight at 37°C with agitation, and the purified genomic DNA was collected from 1 mL of culture using a commercially available kit (ArchivePure DNA cell/tissue kit; 5 Prime, Hilden, Germany). Sequencing was performed by the Microbial Genome Sequencing Center (Pittsburgh, PA, USA). Library prep was conducted using a modified version of the Nextera DNA kit with no size selection, and the library was sequenced on the NextSeq 500 platform (13). A total of 3,331,952 raw read pairs of 150 bp were generated. Raw sequencing reads were filtered to remove contaminating *phiX* reads using BBDuk of the BBTools software suite (https://sourceforge.net/projects/bbmap/). The raw reads were also filtered to remove contaminating Illumina adapter sequences and quality trimmed using Trimmomatic v0.36 (14). All software used the default values unless otherwise noted. The resulting filtered reads were assembled using SPAdes v3.14.1 (15). The assemblies were then filtered to contain only contigs longer than 500 bp with a k-mer coverage of ≥5×. The genome sequence consists of 102 contigs with an *N*_50_ value of 98,144 bp and a sequencing coverage of 110.2×. The resulting assembled genome size was 4,535,289 bp with a G + C content of 40.44%.

### LPS purification

LPS was isolated using the double hot phenol method. Briefly, 2 L of bacterial culture were harvested by centrifugation and resuspended in 90% phenol:endotoxin-free water (1:1 vol/vol) and subsequently incubated at 65°C for 1 h. After centrifugation, the aqueous phase was isolated from the two-phase solution (repeated three times total, pooling the aqueous phase each time) and dialyzed against deionized water using 1 kD MWCO tubing for 36 h, followed by freezing and lyophilization. The lyophilized product was resuspended in 20 mM Tris-HCl pH 8.4, supplemented with 2 mM MgCl_2_ and digested using 500 units of Benzonase and 100 µg/mL DNase I for 2 h at 37°C. The pH was subsequently adjusted to 7.4 and further digested with 100 µg/mL Proteinase K for 2 h at 37°C. Water-saturated phenol was added, followed by centrifugation and collection of the aqueous phase from the two-phase solution, dialyzed as stated above, and frozen before lyophilization. Further isolation of LPS was performed by serial washes in chloroform:methanol (2:1 vol/vol) as described (16). Subsequently, the LPS was separated from contaminating lipoproteins as previously described (17) by resuspension in 0.2% TEA and 0.5% DOC, followed by the addition of 37°C water-saturated phenol and isolation of the aqueous phase from the two-phase solution. Finally, the purified LPS was precipitated by the addition of cold 100% ethanol and 30 mM sodium acetate followed by incubation for 18 h at −20°C. The LPS precipitate was harvested by centrifugation, washed in cold 100% ethanol, resuspended in endotoxin-free water, frozen, and lyophilized.

### Mass spectrometric (MS) and tandem MS analysis of lipid A structure

Prior to LPS extraction, bacterial biomass was isolated from the harvested pellet and resuspended in 200 µL endotoxin-free water. Following that resuspension, the bacterial pellet was harvested by centrifugation and subsequently resuspended in 10 µL of endotoxin-free water to be used for analysis. MS analysis was conducted using the Fast lipid A analysis Technique, known as FLAT, as previously described (18), and tandem MS analysis was conducted using the modified protocol, known as FLAT^n^ (19). Briefly, 1 µL of citrate buffer (200 mM citric acid, 100 mM trisodium citrate, pH 3.5) mixed with 1 µL of bacterial resuspension was deposited onto the sample spot of an ITO slide. The plate was incubated in a humidified, closed glass chamber for 30 min at 110°C. After heating, the ITO slide was removed from the chamber, cooled, washed with deionized water, and left to dry on the laboratory bench. Analysis was then performed using a Bruker MALDI trapped ion mobility spectrometry Time-of-Flight (timsTOF) Mass Spectrometer equipped with a dual ESI/MALDI source with a SmartBeam 3D 10 KHz frequency tripled Nd:YAG laser (355 nm). The system was operated in “qTOF” mode (tims deactivated). Ion transfer tuning used the following parameters: Funnel 1 RF: 440.0 Vpp, Funnel 2 RF: 490.0 Vpp, Multipole RF 490.0 Vpp, is CID Energy: 0.0 eV, and Deflection Delta: −60.0 V. The quadrupole used the following values for MS mode: Ion Energy: 4.0 eV and Low Mass: 700.00 *m/z*. Collision cell activation of ions used the following values for MS mode. Collision Energy: 9.0 eV and Collision RF: 3,900.0 Vpp. In tandem MS mode, the precursor ion was chosen by inputting targeted *m/z* values including two digits beyond the decimal point. Typical isolation width and collision energy were set to 4–6 *m/z* and 100–110 eV, respectively. Focus Pre-TOF used the following values. Transfer time 110.0 µs and pre pulse storage 9.0 µs. Agilent ESI Tune Mix was used to perform calibration. MALDI parameters in qTOF mode were optimized to maximize intensity by tuning ion optics, laser intensity, and laser focus. All spectra were collected at a laser diameter of 104 µm with beam scan on using 800 laser shots per spot and 70% and 80% laser power, respectively. Both MS and tandem MS data were collected in negative ion mode. In all cases, a matrix of 10 mg/mL Norharmane dissolved in chloroform:methanol (2:1 vol/vol) was used for lipid A detection. All MS and tandem MS data were visualized using mMass version 5.5.0 (20). Peak picking was conducted in mMass using the following parameters: S/N threshold: 3.0, absolute intensity threshold: 1.0, relative intensity threshold: 1.0%, picking height: 50, apply baseline, and apply smoothing. Identification of all fragment ions was determined based on ChemDraw Ultra version 10.0.

### Fatty acid methyl ester (FAME) analysis

Lipid A fatty acid content was measured via gas chromatography coupled to flame ionization detection (GC-FID) after acid hydrolysis, methylation, and hexane extraction. For sample preparation, cleavage of the ester and amide bonds linking lipid A acyl chains to the glucosamine backbone was achieved by incubating hot phenol-water extracted LPS in the presence of 2 M methanolic HCl at 90°C for 18 h, subsequently converting fatty acids into fatty acid methyl esters. These fatty acid methyl esters were then extracted using hexane and analyzed via GC-FID. Internal standard quantitation was achieved using a C15 standard. For sample analysis, GC-FID separation and detection were carried out on a Shimadzu GC-MS QP2010 Ultra twin-line machine (one to MS and one to FID), where both lines were equipped with a DB-5ms (30 m × 0.25 mm i.d. × 0.25 µm film thickness, 5% phenyl–95% dimethylpolysiloxane) analytical column from Agilent. The GC instrument was equipped with a split/splitless inlet. Inert split/splitless liners were used. For analysis, 1 µL of sample was injected into the FID line inlet in split mode at an injection temperature of 250°C. The following GC temperature program was used: 80°C (hold 1 min), 25°C/min ramp to 160°C (hold 1 min), 10°C/min ramp to 265°C, 1°C/min ramp to 270°C (hold 1 min), and 10°C/min ramp to 295°C (hold 1 min). Ultra-high purity helium (UHP300) was used as a carrier gas, maintained at a constant pressure of 110.7 kPa, translating to a linear velocity of 31.3 cm/s. The FID was maintained at a temperature of 325°C with a makeup flow of 30 mL/min Helium (UHP300), 40 mL/min compressed hydrogen, and 400 mL/min compressed air. For data evaluation, LabSolutions LC/GC version 5.99 (Shimadzu) was utilized.

### NF-κB reporter assay

HEK293T cells stably expressing TLR4/MD-2/CD-14 and a secreted embryonic alkaline phosphatase (SEAP) gene under the control of the NF-κB promoter were obtained from Invivogen (cat# hkb-htlr4 and hkb-mtlr4). Sterile, cell culture-treated, 96-well flat-bottom plates were seeded with 6 × 10^4^ cells/well and incubated for 18 h at 37°C with 5% CO_2_. Cells were stimulated with LPS ranging from 10^−1^ to 10^3^ ng/mL, followed by incubation at 37°C with 5% CO_2_ for 18 h. The Quanti-Blue (QB) reagent was prepared according to the manufacturer’s protocols (Invitrogen cat# rep-qbs). Briefly, 1 mL of QB buffer was mixed with 1 mL of QB reagent top stock and dissolved in 100 mL total of sterile endotoxin-free water. After the stimulation, 20 µL of cell supernatant was mixed with 180 µL of QB reagent in 96-well plates and incubated for 30 min or until the highest absorbance reading at 630 nm reached 1.5. The percent of relative NF-κB activation was determined by normalizing to the absorbance values of LPS stimulation at saturating conditions (10^3^ ng/mL).

## RESULTS

### *P. damselae* subsp. *damselae* strain SF21 was isolated from a nurse shark stool

For this study, stool from a nurse shark was collected and cultured at 37°C to evaluate bacterial growth at human physiological temperature. Interestingly, only a single organism grew at this temperature, suggesting adaptation for proliferation at temperatures exceeding the shark’s internal body temperature, which is likely that of the ambient water (~28°C). Genomic sequencing identified the organism as *Photobacterium damselae*, a known marine pathogen and an opportunistic pathogen in humans (21–24). This species comprises two subspecies, *piscicida* and *damselae* (25), and because only subsp. *damselae* has been shown to grow at 37°C (26), consistent with our genomic sequencing data, the isolate was identified as *P. damselae* subsp. *damselae*. For this study, the isolate was named SF21 (SF for shark feces and 21 for the year we first isolated it).

### SF21 generates pEtN-modified lipid A

**Fig 1:**
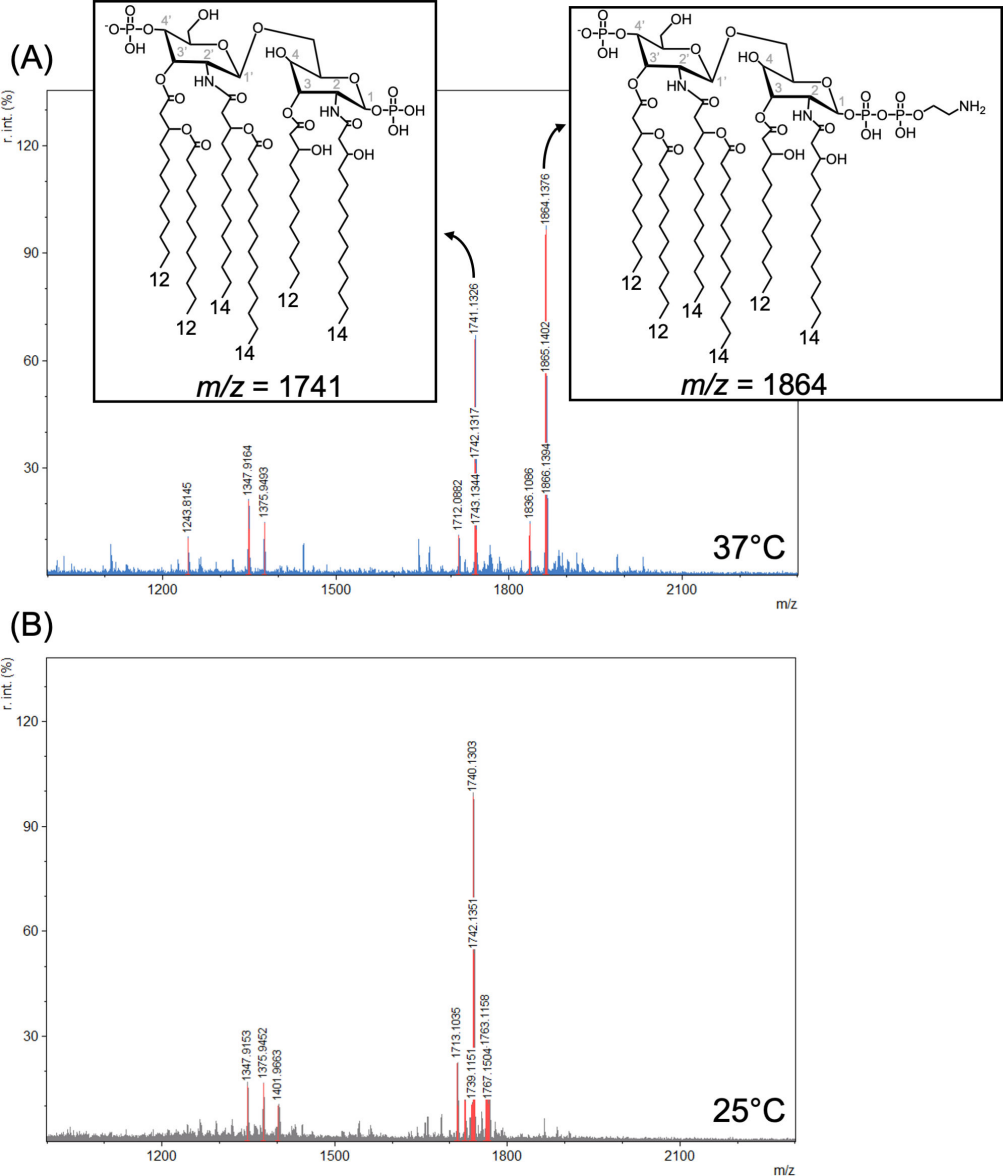
MALDI-TOF MS analysis of lipid A from SF21 grown at 37°C (**A**) or 25°C (**B**). The determined lipid A structures are depicted. Carbon positions along the diglucosamine backbone are denoted in gray. Acyl chain lengths are denoted.

Given that no other organism from the shark stool grew at 37°C and that this temperature is reported to be a stressful condition for *Pdd* (27), we reasoned that the lipid A structure, which is a key structural component of the outer membrane, could contain unique features. We then cultured SF21 at 37°C and subsequently analyzed its lipid A structure via MALDI-TOF MS. Ions at *m/z* = 1,741 and *m/z* = 1,864 suggested two predominant diphosphorylated hexa-acylated lipid A structures, differing only by the absence or presence of a pEtN modification (∆*m/z* = 123), respectively (Fig. 1A). The presence of the pEtN modification was supported by genetic evidence of three EptA orthologs present in the genome (all >96% similar to the pEtN-transferases within *E. coli*). To confirm the location of the pEtN-modification, we employed tandem MS analysis. Upon fragmenting the two ions of interest (*m/z* = 1,741 and 1,864), the pEtN-modified structure generated a unique ion at *m/z* = 805, which confirmed its location at the 1-carbon position (Fig. S1 and S2). Since 37°C is likely a higher temperature than this isolate encounters in its natural environment, we determined the lipid A structure after growth at 25°C, a temperature more reflective of the internal body temperature of a shark (28). At this temperature, only the peak at *m/z* = 1,741 was present (Fig. 1B), suggesting that pEtN-modification may be a stress response for SF21 when grown at temperatures above its normal environment.

### SF21 possesses intrinsic antimicrobial resistance

**Fig 2:**
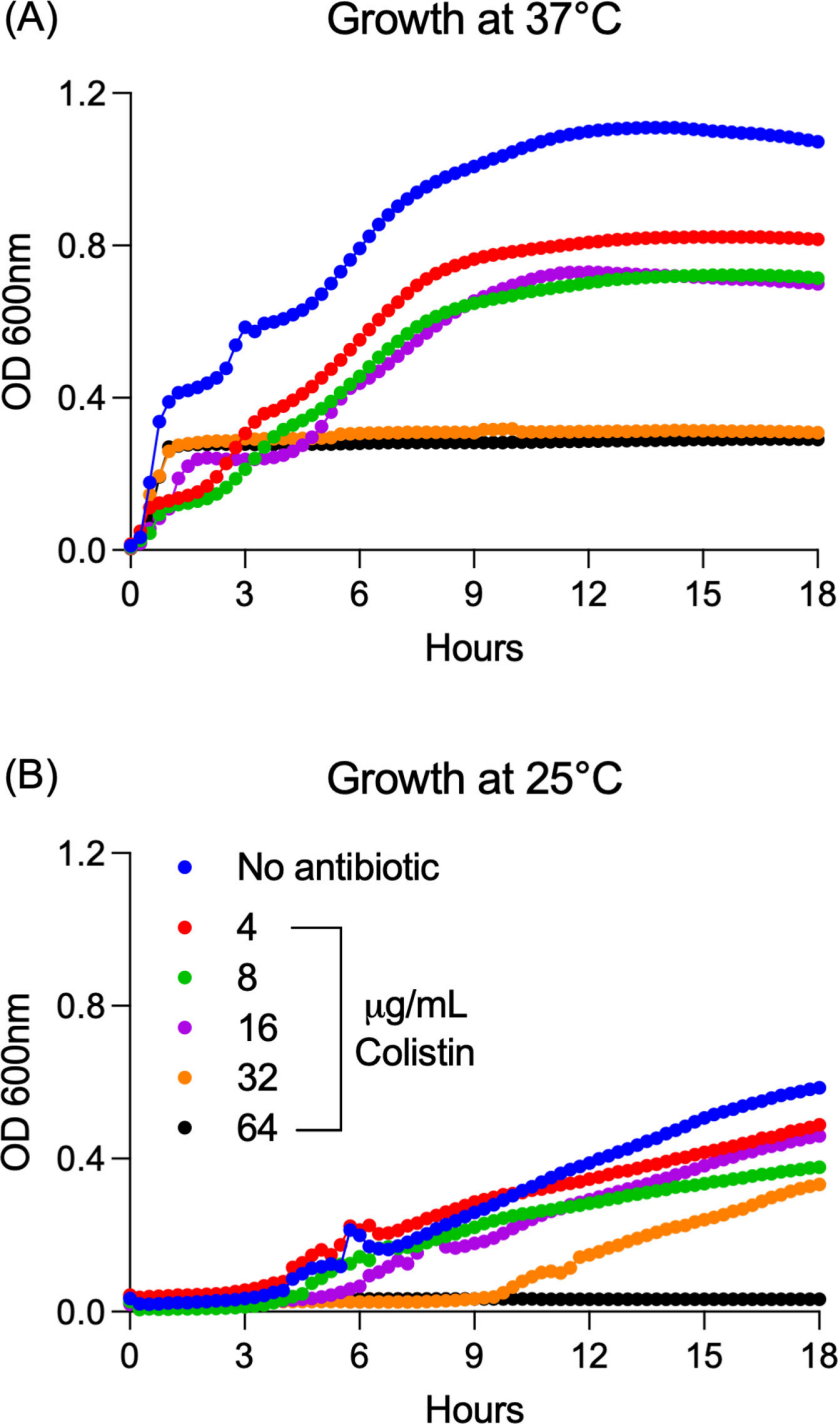
SF21 grown in the absence or presence of increasing concentrations of colistin at 37°C (**A**) or 25°C (**B**). Growth was monitored by the Cerillo instrument, taking OD_600 nm_ measurements every 15 min. Error bars are omitted for clarity. Dots represent the average of three biological replicates. Colors correspond to the same concentrations of colistin for both (**A**) and (**B**).

Typically, pEtN modification is induced by CAMPs such as colistin, which target the anionic phosphates of lipid A (8); however, our MS analysis demonstrated that elevated temperature could elicit this modification without prior CAMP exposure. To determine if SF21 was intrinsically resistant to CAMPs, we cultured SF21 in media containing escalating colistin concentrations at both 37°C and 25°C. Despite slower growth at 25°C, SF21 exhibited clear resistance at both temperatures, with a minimum inhibitory concentration of 32 µg/mL at 37°C (Fig. 2A) and 64 µg/mL at 25°C (Fig. 2B), although growth at 25°C was delayed in the presence of 32 µg/mL colistin. The absence of a pEtN-modified peak in cultures of SF21 grown without colistin at 25°C (Fig. 1B), but the observed resistance to colistin under these conditions (Fig. 2B) suggests that pEtN modification occurs rapidly in this strain upon CAMP exposure or temperature shift.

### SF21 selectively modifies its acyl chain at the 2′-carbon position at cold temperatures

**Fig 3:**
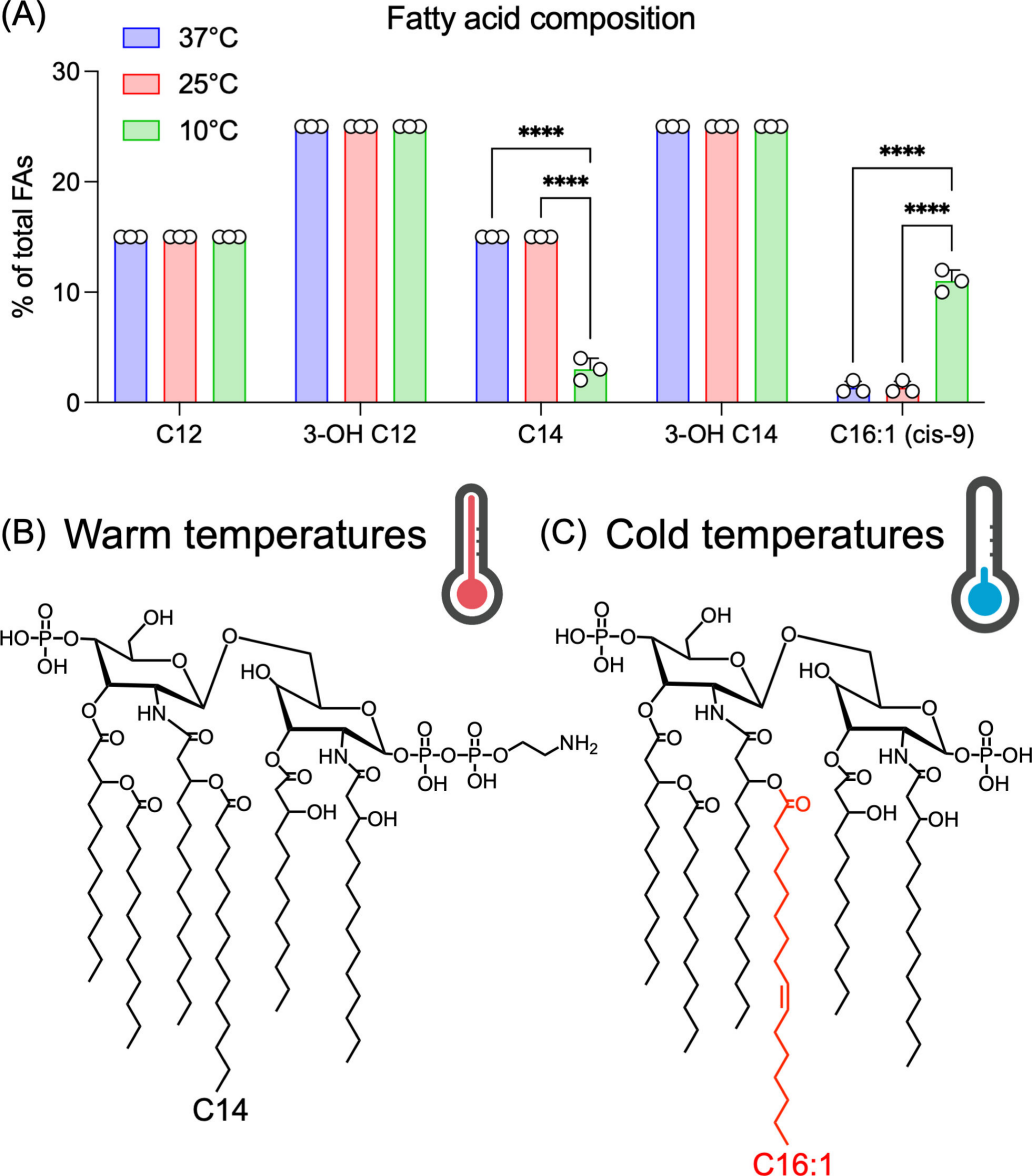
GC-FID fatty acid compositional analysis of SF21 lipid A following growth of SF21 at 37°C, 25°C, and 10°C. (**A**) The abundance of each fatty acid normalized to the total abundance of all fatty acids identified. Representative lipid A structures for SF21 upon growth in (**B**) warm conditions (25°C and 37°C) and (**C**) cold conditions (10°C). Data points represent quantitation from biological triplicates. Statistical significance was determined by ordinary two-way ANOVA with Tukey’s multiple comparison test, with a single pooled variance. **** represent *P* values ≤ 0.0001.

As we have shown, SF21 modifies its lipid A with pEtN to support growth under stress. Thus, we next examined whether it was capable of additional stress-associated lipid A structural adaptations. Based on genetic evidence for the presence of LpxP (97% similarity to the lipid A palmitoleoyl transferase from *Salmonella*), we compared the fatty acid composition of SF21 grown under cold conditions (10°C) to that of warm conditions (25°C and 37°C). Gas chromatography analysis of fatty acid methyl esters detected the expected lipid A-derived fatty acids at 37°C and 25°C (Fig. 1); however, growth at 10°C resulted in a distinct shift in fatty acid composition, characterized by loss of C14 and the appearance of C16:1 (cis-9) (Fig. 3A). Thus, in addition to pEtN modification, SF21 also modulates its lipid A fatty acids to potentially preserve membrane stability, incorporating C14 under warm (Fig. 3B) and C16:1 under cold conditions (Fig. 3C).

### SF21 LPS stimulates human versus mouse TLR4/MD-2 differently than *E. coli* LPS

**Fig 4:**
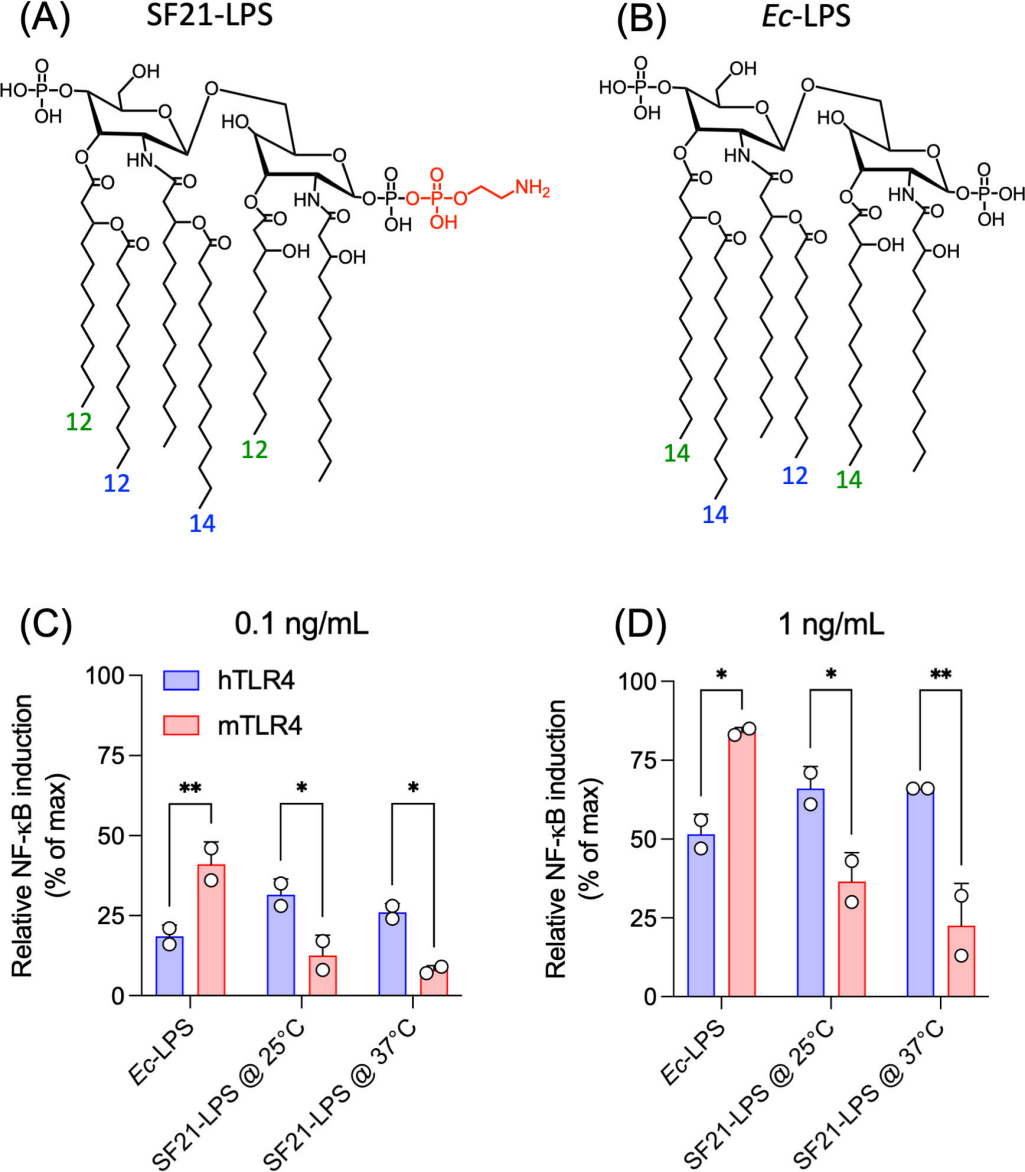
Comparison of SF21 and *E. coli* LPS recognition by different orthologs of TLR4/MD-2. The lipid A structure of (**A**) SF21 LPS (SF21-LPS) and (**B**) *E. coli* LPS (*Ec*-LPS). The differences between the two structures are highlighted in corresponding colors to assist in identifying the differences. Stimulation of NF-κB reporter cells expressing the human (hTLR4) or mouse (mLTR4) orthologs of TLR4/MD-2/CD-14 with (**C**) 0.1 ng/mL LPS or (**D**) 1 ng/mL LPS. SF21-LPS was purified from large-scale cultures grown at either 25°C or 37°C. Data are shown as NF-κB induction normalized to LPS stimulation at saturating conditions (1 µg/mL). Data points represent biological duplicates. Statistical significance was determined by ordinary two-way ANOVA with Šídák’s multiple comparison test, with a single pooled variance. * and ** represent *P* values of ≤0.05 and ≤0.01, respectively.

It has been shown previously that diphosphorylated hexa-acylated lipid A signals stronger through the mouse TLR4/MD-2 complex than the human ortholog (29–32); therefore, we determined if SF21 lipid A modified with pEtN modification could alter the distinct signaling patterns usually observed between the mouse and human TLR4/MD-2 receptors. NF-κB reporter cells expressing either the human or mouse TLR4/MD-2 receptor complexes were stimulated with purified SF21 LPS (SF21-LPS) obtained from cultures grown at 25°C or 37°C (Fig. 4A) and compared with *E. coli* LPS (Ec-LPS), which contains a hexa-acylated lipid A lacking pEtN modification (Fig. 4B). At both concentrations tested, Ec-LPS elicited significantly stronger signaling through mouse (mTLR4) than human (hTLR4) reporter cells, whereas SF21-LPS produced the opposite pattern, signaling more robustly through hTLR4 reporter cells instead (Fig. 4C and D). These data suggest that pEtN-modification at the 1-carbon position may play a role in the differential response to LPS across human and murine TLR4/MD-2 and warrants future studies to dissect this observation.

## DISCUSSION

Here, we characterize a *P. damselae* subsp. *damselae* isolate, SF21, that constitutively synthesizes pEtN-modified lipid A at 37°C and rapidly induces this modification upon CAMP exposure. Prior transcriptomic analyses of other *Pdd* strains did not identify *eptA* as differentially regulated following temperature shifts (27, 33), which may reflect strain-specific variation or indicate that pEtN transferase activity is not governed solely at the transcriptional level. Although pEtN modification likely contributes to colistin resistance, antimicrobial resistance in SF21 is probably multifactorial and may involve additional mechanisms not examined here.

Mechanistically, pEtN modification is assumed to mask the negatively charged phosphates of lipid A and disrupt CAMP-mediated membrane binding (8); however, it has also been reported to restrict membrane permeability (34). Based on these established roles, our data suggest that pEtN modification supports bacterial adaptation to harsh conditions, perhaps explaining how SF21 was capable of growth at 37°C while other shark stool bacteria, better suited for growth at 28°C, were unable to. The specific mechanism, whether involving membrane stabilization, enhanced fluidity, or both, remains to be determined.

In addition to pEtN modification, SF21 exhibited additional lipid A structural changes that supported growth across varied temperatures. Growth at 10°C induced a cold-shock response characterized by the incorporation of an unsaturated fatty acid, likely enhancing membrane fluidity. This is in line with prior studies that show cold-induced incorporation of unsaturated C16:1 into lipid A by other gram-negative bacteria (3, 35, 36). In contrast, a different *Pdd* strain has been shown to incorporate distinct saturated fatty acids at higher growth temperatures (27). This, combined with our data, suggests that *Pdd* can remodel its membrane lipids (including lipid A) upon growth at varied temperatures to accommodate various environmental stresses.

The acyl chain configuration of SF21 lipid A closely resembles that of *E. coli*, differing primarily in the positioning of the C12 and C14 secondary acyl chains and in the presence of shorter C12, rather than C14, chains at the 3 and 3′ positions of the glucosamine backbone (Fig. 4A and B). Unlike *E. coli*, SF21 exhibits a pEtN modification at the 1-carbon of lipid A (Fig. 4A and B), a difference that surprisingly reversed the prototypical LPS response by human versus mouse TLR4/MD-2. In contrast to *E. coli* LPS, which preferentially activates mouse TLR4/MD-2 (29–32), SF21 LPS triggered stronger signaling through the human ortholog of TLR4/MD-2 (Fig. 4C and D). Although differences in acyl chain configuration may contribute, our data, together with prior studies (37, 38), suggests that pEtN modification could be the primary driver of altered species-specific TLR4/MD-2 recognition.

These findings raise questions about the functional role of lipid A pEtN modification. Whether SF21 synthesizes this modification to alter TLR4 immunogenicity, for membrane stability, or to enable antimicrobial resistance remains unclear. It is also unknown whether these lipid A features are conserved across *Pdd* strains or unique to this isolate. Lipid A pEtN modification is widespread among gram-negative bacteria where it serves diverse functions (extensively reviewed here [39]), suggesting its benefit to bacterial fitness may not be through one particular mechanism. One well-studied example is *E. coli* Nissle 1917 (40), which constitutively synthesizes pEtN-modified lipid A; however, despite its well-documented anti-inflammatory properties (40, 41) and probiotic potential (42), the evolutionary advantage of this persistent modification remains unclear.

There is evidence that CAMPs represent an evolutionarily ancient defense strategy, as many bacteria produce CAMP-like molecules (such as bacteriocins) for interbacterial competition (43). Accordingly, pEtN modification of lipid A may have arisen early as a mechanism to counter this molecular pressure. The prevalence of this modification in natural environments, particularly in the apparent absence of antimicrobial pressure, remains to be studied further. Defining its distribution and context will be critical to understanding whether pEtN modification primarily supports pathogenicity, environmental persistence, or both.

## Supplementary Material

Reviewer comments

## Data Availability

The data sets generated and analyzed from this study are available from the corresponding author on reasonable request. This Whole Genome Shotgun project has been deposited at DDBJ/ENA/GenBank under accession number JBUEDZ000000000. The version described in this paper is JBUEDZ010000000.

## SUPPLEMENTAL MATERIAL

Supplemental figures spectrum.00662-26-s0001.pdf Fig. S1 and S2.

OPEN PEER REVIEW reviewer-comments.pdf An accounting of the reviewer comments and feedback.

ASM does not own the copyrights to Supplemental Material that may be linked to, or accessed through, an article. The authors have granted ASM a non-exclusive, world-wide license to publish the Supplemental Material files. Please contact the corresponding author directly for reuse.
