# Supplementary material for: Phosphoethanolamine-modified lipid A enables antimicrobial resistance and atypical signaling properties in the marine pathogen P. damselae subsp. damselae
Source: Microbiol Spectr. 2026 Jul 14;14(8):e00662-26. doi: 10.1128/spectrum.00662-26 (PMC13436394; doi:10.1128/spectrum.00662-26)
Supplement: Supplementary file 1 [file reviewer-comments.pdf]

AMERICAN SOCIETY FOR MICROBIOLOGY | Microbiology Spectrum

Phosphoethanolamine-modified lipid A enables antimicrobial resistance and atypical signaling properties in the marine pathogen *P. damselae* subsp. *damselae*

Matthew Sherman, Hyojik Yang, David Rasko, Joshua Kolb, Anuj Joshi, Udara Mendis, Helen Dooley, Alison Scott, David Goodlett, and Robert Ernst

Corresponding Author(s): Robert Ernst, University of Maryland, Baltimore

Review Timeline:

Submission Date:	February 26, 2026
Editorial Decision:	April 14, 2026
Revision Received:	May 8, 2026
Accepted:	May 27, 2026

Editor: Zhi Ruan

Reviewer(s): Disclosure of reviewer identity is with reference to reviewer comments included in decision letter(s). The following individuals involved in review of your submission have agreed to reveal their identity: Carlos R. Osorio (Reviewer #1); Xose Manuel Matanza Fente (Reviewer #2)

Transaction Report:

(Note: With the exception of the correction of typographical or spelling errors that could be a source of ambiguity, letters and reports are not edited. The original formatting of letters and referee reports may not be reflected in this compilation.)

DOI: https://doi.org/10.1128/spectrum.00662-26

Re: Spectrum00662-26 (Phosphoethanolamine-modified lipid A enables antimicrobial resistance and atypical signaling properties in the marine pathogen *P. damselae* subsp. *damselae*)

Dear Dr. Robert K Ernst:

Thank you for the privilege of reviewing your work. Below you will find my comments, instructions from the Spectrum editorial office, and the reviewer comments.

Please return the manuscript within 60 days; if you cannot complete the modification within this time period, please contact me. If you do not wish to modify the manuscript and prefer to submit it to another journal, notify me immediately so that the manuscript may be formally withdrawn from consideration by Spectrum.

Revision Guidelines
To submit your modified manuscript, log into the submission site at https://spectrum.msubmit.net/cgi-bin/main.plex. Go to Author Tasks and click the appropriate manuscript title to begin. The information you entered when you first submitted the paper will be displayed; update this as necessary. Note the following requirements:

• Upload point-by-point responses to the issues raised by the reviewers in a file named "Response to Reviewers," NOT in your cover letter.
• Upload a compare copy of the manuscript (without figures) as a "Marked-Up Manuscript" file.
• Upload a clean .DOC/.DOCX version of the revised manuscript and remove the previous version.
• Each figure must be uploaded as a separate, editable, high-resolution file (TIFF or EPS preferred), and any multipanel figures must be assembled into one file.
• Any supplemental material intended for posting by ASM should be uploaded with their legends separate from the main manuscript. You can combine all supplemental material into one file (preferred) or split it into a maximum of 10 files with all associated legends included.

For complete guidelines on revision requirements, see our Submission and Review Process webpage. Submission of a paper that does not conform to guidelines may delay acceptance of your manuscript.

Data availability: ASM policy requires that data be available to the public upon online posting of the article, so please verify all links to sequence records, if present, and make sure that each number retrieves the full record of the data. If a new accession number is not linked or a link is broken, provide Spectrum production staff with the correct URL for the record. If the accession numbers for new data are not publicly accessible before the expected online posting of the article, publication may be delayed; please contact production staff (Spectrum@asmusa.org) immediately with the expected release date.

Publication Fees: For information on publication fees and which article types are subject to charges, visit our website. If your manuscript is accepted for publication and any fees apply, you will be contacted separately about payment during the production process; please follow the instructions in that e-mail. Arrangements for payment must be made before your article is published.

ASM Membership: Corresponding authors may join or renew ASM membership to obtain discounts on publication fees. Need to upgrade your membership level? Please contact Customer Service at Service@asmusa.org.

The ASM Journals program strives for constant improvement in our submission and publication process. Please tell us how we can improve your experience by taking this quick Author Survey.

Thank you for submitting your paper to Spectrum.

Sincerely,

Professor Zhi Ruan
Editor
Microbiology Spectrum

Reviewer #1 (Comments for the Author):

The manuscript by Sherman et al. presents a structural and functional characterization of lipid A from Photobacterium damselae subsp. damselae isolated from nurse shark feces, focusing on (i) phosphoethanolamine (pEtN) modification, (ii) intrinsic colistin resistance, (iii) temperature-dependent acyl chain remodeling, and (iv) atypical differential activation of human versus murine TLR4/MD-2.
The study is technically sound, clearly written, and well aligned with the scope of Microbiology Spectrum, particularly in the

areas of host-pathogen interactions, membrane lipid remodeling, and antimicrobial resistance. The use of complementary approaches (MALDI-TOF/FLAT lipid A profiling, FAME analysis, MIC assays, and NF-κB reporter systems) is a notable strength. Importantly, the observation that a hexa-acylated, pEtN-modified lipid A preferentially activates hTLR4 over mTLR4 is intriguing and potentially impactful.
That said, several conceptual and interpretative issues should be addressed or more carefully framed before publication.

Comments
1. Causal Link Between pEtN Modification and Colistin Resistance:
The association between lipid A pEtN modification and intrinsic colistin resistance is largely inferred rather than directly demonstrated. In this regard, other resistance mechanisms (e.g., core oligosaccharide alterations, outer membrane proteins, efflux systems) are not evaluated. At minimum, explicitly acknowledge that colistin resistance may be multifactorial. Ideally, additional genetic or biochemical validation would strengthen the causal link, although this could also be framed as a limitation of the current study.
2. Generalizability of Findings. The study is based on a single isolate (SF21). However, as previous studies have extensively reported, P. damselae subsp. damselae is a highy diverse subspecies. It remains unclear whether the described lipid A features are common among P. damselae subsp. damselae strains or specific to this isolate.
Therefore, this reviewer recommends that authors explicitly acknowledge that conclusions are based on a single strain and discuss the extent to which these findings may (or may not) generalize across the species.
3.- The genome of this strain is supposed to have been sequenced and deposited in GenBank under Bioproject PRJNA1416945. However, when this reviewer tried to retrieve this informnation at the NCBI page, it retrieved no results. It is mandatory that the authors make this genome information available before publication.

Reviewer #2 (Comments for the Author):

In this study, the authors isolated a strain of the Gram-negative bacterium Photobacterium damselae subsp. damselae from the cultivation of shark stools at 37ºC. The authors conducted a lipid A MS analysis and found that cultivation at 37ºC increased the amount of phosphoethanolamine (pEtN) in the lipid A of P. damselae. The presence of pEtN in the lipid A is associated with resistance to colistins, however, the authors found that P. damselae is inherently resistant to colistin E both at 25 and 37ºC. Additionally, the authors show that P. damselae LPS elicits a greater NF-Kb response in human immune cells in comparison to murine cells.
Overall, this is a well-written study but it is difficult to identify its novelties. Whilst experiments show technical validity, the current version of the manuscript lacks a contextualization of the findings in the field. A comparison with previous studies on the role of temperature in the pathobiology of P. damselae is missing. This leads to a poor description of what the current findings mean in relation to what is already known about the research problem. The introduction is well-written, however the results and discussion deserve further details and justification.

Specific comments are attached below:
Introduction: The role of temperature in the pathobiology of P. damselae subsp. damselae has already been studied (https://doi.org/10.1371/journal.pone.0210118; https://doi.org/10.3389/fmicb.2020.01771). None of this is acknowledged in the present manuscript. More importantly, 37ºC has been demonstrated to be itself a stressful condition for Pdd that leads to abnormal morphology, cell death and the activation of stress responses (https://doi.org/10.3389/fmicb.2020.01771). These studies should be cited and discussed in the present manuscript for a proper contextualization in the field.
Line 79. The mechanisms that incorporate pEtN in Gram-negative bacteria do not only include mcr-encoding plasmids. Please include all known mechanisms as this is relevant to this study. For example the role of gene pmrC encoding for EptA, and regulated by PmrAB should be introduced.
Line 96-97. This sentence needs to be rewritten/toned down. In its current form, it suggests that it is specifically identified in this study that Pdd is capable of growing at 37ºC. However, this is a well-known capacity of Pdd previously identified and investigated as a stressful condition (https://doi.org/10.3389/fmicb.2020.01771).
Line 252-255. This is the first introduction of the role of EptA in the incorporation of pEtN in the lipid A in the manuscript. As stated below this information should be included in the introduction for clarity.
Line 255-275. The authors show that the intrinsic resistance to colistin, even at 25ºC suggests that pEtN modification occurs rapidly upon CAMP exposure or temperature shift. Are eptA genes upregulated at 37ºC? Are those identified in previously published transcriptomic studies conducted at 37ºC for P. damselae? This information should be added in the results section or discussion as these has been studied elsewhere and may provide better contextualization.
314-316. Lipid A modifications are well-known mechanisms important for cell survival under stress, including increases in growth temperature (https://doi.org/10.1128/mmbr.00193-23). Have the authors found any role for pEtN in surviving high temperatures in the literature or is this a novelty of the current study? Please clarify in the manuscript.
318-324. The FAME findings should be placed in the context of the literature. Previous authors have already found that P. damselae subsp. damselae modifies its membrane lipid composition at lower temperatures to maintain fluidity, often by incorporating higher levels of unsaturated fatty acids (https://doi.org/10.3389/fmicb.2020.01771). Are there commonalities/contradictions between the current study and already published datasets?

Response to Reviewers

Reviewer #1 comments:

1. Causal Link Between pEtN Modification and Colistin Resistance. The association between lipid A pEtN modification and intrinsic colistin resistance is largely inferred rather than directly demonstrated. In this regard, other resistance mechanisms (e.g., core oligosaccharide alterations, outer membrane proteins, efflux systems) are not evaluated. At minimum, explicitly acknowledge that colistin resistance may be multifactorial. Ideally, additional genetic or biochemical validation would strengthen the causal link, although this could also be framed as a limitation of the current study.

We thank the reviewer for this valid point. On lines 313-314 we discuss that while we believe lipid A pEtN modification enables colistin resistance, there may be other mechanisms that enable antimicrobial resistance which were not investigated in this study.

2. Generalizability of Findings. The study is based on a single isolate (SF21). However, as previous studies have extensively reported, P. damselae subsp. damselae is a highly diverse subspecies. It remains unclear whether the described lipid A features are common among P. damselae subsp. damselae strains or specific to this isolate. Therefore, this reviewer recommends that authors explicitly acknowledge that conclusions are based on a single strain and discuss the extent to which these findings may (or may not) generalize across the species.

This is an insightful comment, and we agree it must be mentioned that only a single isolate was pursued for this study. On lines 347-348 we acknowledge that future investigations of other *P. damselae* subsp. *damselae* (*Pdd*) strains are required to confirm that this is a conserved feature. We also modified our language to be more specific. Instead of broadly referring to our strain as *Pdd*, we refer to it specifically as SF21, indicating that the findings are for this one isolate rather than the genus or subspecies as a whole.

3. The genome of this strain is supposed to have been sequenced and deposited in GenBank under Bioproject PRJNA1416945. However, when this reviewer tried to retrieve this information at the NCBI page, it retrieved no results. It is mandatory that the authors make this genome information available before publication.

We have since published the sequence and it should be available now under the accession JBUEDZ000000000. Thank you for bringing this to our attention.

Reviewer #2 comments:

1. Introduction: The role of temperature in the pathobiology of P. damselae subsp. damselae has already been studied (https://doi.org/10.1371/journal.pone.0210118; https://doi.org/10.3389/fmicb.2020.01771). None of this is acknowledged in the present manuscript. More importantly, 37ºC has been demonstrated to be itself a stressful condition for Pdd that leads to abnormal morphology, cell death and the activation of stress responses (https://doi.org/10.3389/fmicb.2020.01771). These studies should be cited and discussed in the present manuscript for a proper contextualization in the field.

We thank the reviewer for bringing this to our attention. These papers have provided valuable insight into our findings. We have incorporated these references where appropriate in the manuscript (lines 250, 311, and 331).

2. Line 79. The mechanisms that incorporate pEtN in Gram-negative bacteria do not only include mcr-encoding plasmids. Please include all known mechanisms as this is relevant to this study. For example, the role of gene pmrC encoding for EptA, and regulated by PmrAB should be introduced.

This is a very important point, and we agree should be clearer. On lines 77-81 we have revised the language to explicitly state that pEtN-modification is through either PmrAB-mediated EptA activation or expression of pEtN-transferases via mcr-encoding plasmids.

3. Line 96-97. This sentence needs to be rewritten/toned down. In its current form, it suggests that it is specifically identified in this study that Pdd is capable of growing at 37ºC. However, this is a well-known capacity of Pdd previously identified and investigated as a stressful condition (https://doi.org/10.3389/fmicb.2020.01771).

We agree that the statement in its current form may misrepresent the novelty of *Pdd* growth at 37°C. On lines 98-100 the sentence has been rewritten to be more precise with the language and statement we are trying to convey.

4. Line 252-255. This is the first introduction of the role of EptA in the incorporation of pEtN in the lipid A in the manuscript. As stated below this information should be included in the introduction for clarity.

We thank the reviewer for mentioning the omission of *eptA* when pEtN-modification is first discussed in the introduction. On lines 77-79 in the introduction, we include an explanation that *eptA* (also known as *pmrC*) encodes a pEtN-transferase responsible for executing this modification.

5. Line 255-275. The authors show that the intrinsic resistance to colistin, even at 25ºC suggests that pEtN modification occurs rapidly upon CAMP exposure or temperature shift. Are eptA genes upregulated at 37ºC? Are those identified in previously published transcriptomic studies conducted at 37ºC for P. damselae? This information should be added in the results section or discussion as these have been studied elsewhere and may provide better contextualization.

In both transcriptomic studies examining the effect of temperature shift on *Pdd*, *eptA* was not found as a differentially transcribed gene. On lines 310-313 we address this by suggesting that pEtN-modification activity may not be controlled at the transcript level or that the strain characterized in this study differs from those used in the transcriptomic analyses.

6. Line 314-316. Lipid A modifications are well-known mechanisms important for cell survival under stress, including increases in growth temperature (https://doi.org/10.1128/mmbr.00193-23). Have the authors found any role for pEtN in surviving high temperatures in the literature or is this a novelty of the current study? Please clarify in the manuscript.

After searching the literature, we find no reports that pEtN-modification alone enables survival at high temperatures. While the data in our study supports this idea, we are careful with our wording not to make that claim since further experiments would be needed to be prove this. On lines 319-323 we speculate that pEtN-modification may enable growth at the higher temperature but do not explicitly state that we our data shows this directly.

7. Line 318-324. The FAME findings should be placed in the context of the literature. Previous authors have already found that P. damselae subsp. damselae modifies its membrane lipid composition at lower temperatures to maintain fluidity, often by incorporating higher levels of unsaturated fatty acids (https://doi.org/10.3389/fmicb.2020.01771). Are there commonalities/contradictions between the current study and already published datasets?

The cited paper conducted FAME on total membrane lipids of *Pdd* grown at 25°C and 37°C and found the distribution of saturated and unsaturated fatty acids in the membrane changed with temperature. Specifically, they show that certain saturated fatty acids are incorporated into membranes at the higher temperature. Our FAME analysis of lipid A-specific acyl chains showed a greater incorporation of a specific unsaturated fatty acid into lipid A when grown at the lowest temperature tested (10°C). On lines 329-333 we cite the paper mentioned and explain that collectively our data and theirs suggests that *Pdd* can remodel its membrane lipids when grown at various temperatures.

Re: Spectrum00662-26R1 (Phosphoethanolamine-modified lipid A enables antimicrobial resistance and atypical signaling properties in the marine pathogen *P. damselae* subsp. *damselae*)

Dear Dr. Robert K Ernst:

Your manuscript has been accepted, and I am forwarding it to the ASM production staff for publication. Your paper will first be checked to make sure all elements meet the technical requirements. ASM staff will contact you if anything needs to be revised before copyediting and production can begin. Otherwise, you will be notified when your proofs are ready to be viewed.

Data Availability: ASM policy requires that data be available to the public upon online posting of the article, so please verify all links to sequence records, if present, and make sure that each number retrieves the full record of the data. If a new accession number is not linked or a link is broken, provide production staff with the correct URL for the record. If the accession numbers for new data are not publicly accessible before the expected online posting of the article, publication may be delayed; please contact ASM production staff immediately with the expected release date.

Publication Fees: For information on publication fees and which article types have charges, please visit our website. We have partnered with Copyright Clearance Center (CCC) to collect author charges. If fees apply to your paper, you will receive a message from no-reply@copyright.com with further instructions. For questions related to paying charges through RightsLink, please contact CCC at ASM_Support@copyright.com or toll free at +1-877-622-5543. CCC makes every attempt to respond to all emails within 24 hours.

ASM Membership: Corresponding authors may join or renew ASM membership to obtain discounts on publication fees. Need to upgrade your membership level? Please contact Customer Service at Service@asmusa.org.

Embargo Policy: A press release may be issued as soon as the manuscript is posted on the Spectrum Latest Articles webpage. The corresponding author will receive an email with the subject line "ASM Journals Author Services Notification" when the article is available online.

The ASM Journals program strives for constant improvement in our submission and publication process. Please tell us how we can improve your experience by taking this quick Author Survey.

Thank you for submitting your paper to Spectrum.

Sincerely,

Professor Zhi Ruan
Editor
Microbiology Spectrum

Reviewer #1 (Comments for the Author):

The minor concerns raised by this reviewer have been satisfactorily addressed in the revised manuscript version

Reviewer #2 (Comments for the Author):

All points raised by this reviewer have been answered and resolved. I do not have further comments.
