# Supplementary material for: Phosphoethanolamine-modified lipid A enables antimicrobial resistance and atypical signaling properties in the marine pathogen P. damselae subsp. damselae
Source: Microbiol Spectr. 2026 Jul 14;14(8):e00662-26. doi: 10.1128/spectrum.00662-26 (PMC13436394; doi:10.1128/spectrum.00662-26)
Supplement: Supplementary file 2 [file spectrum.00662-26-s0001.pdf]

Supplemental Figures

Phosphoethanolamine-modified lipid A enables antimicrobial resistance and atypical signaling properties in the marine pathogen *P. damselae* subsp. *damselae*

Matthew E. Sherman[1], Hyojik Yang[1], David A. Rasko[1], Joshua Kolb[1], Anuj Joshi[2], Udara Mendis[3], Helen Dooley[4], Alison J. Scott[1], David R. Goodlett[2], Robert K. Ernst[1]*

[1]Department of Microbial Pathogenesis, University of Maryland-Baltimore, Baltimore, MD 21201, United States

[2]Department of Biochemistry and Microbiology, University of Victoria, Victoria, British Columbia, V8W 2Y2, Canada

[3]Department of Bioengineering, University of Maryland-College Park, College Park, MD 20742, United States

[4]Department of Microbiology and Immunology, University of Maryland School of Medicine, Institute of Marine and Environmental Technology, Baltimore, MD 21202, United States

*Corresponding author: rkernst@umaryland.edu

650 W. Baltimore St, Rm 9205

Baltimore, MD 21201

(P): 410-706-3622

Table of contents

Figure S1: Tandem MS of the Lipid A structure without pEtN-modification 3

Figure S2: Tandem MS of the Lipid A structure with pEtN-modification 4

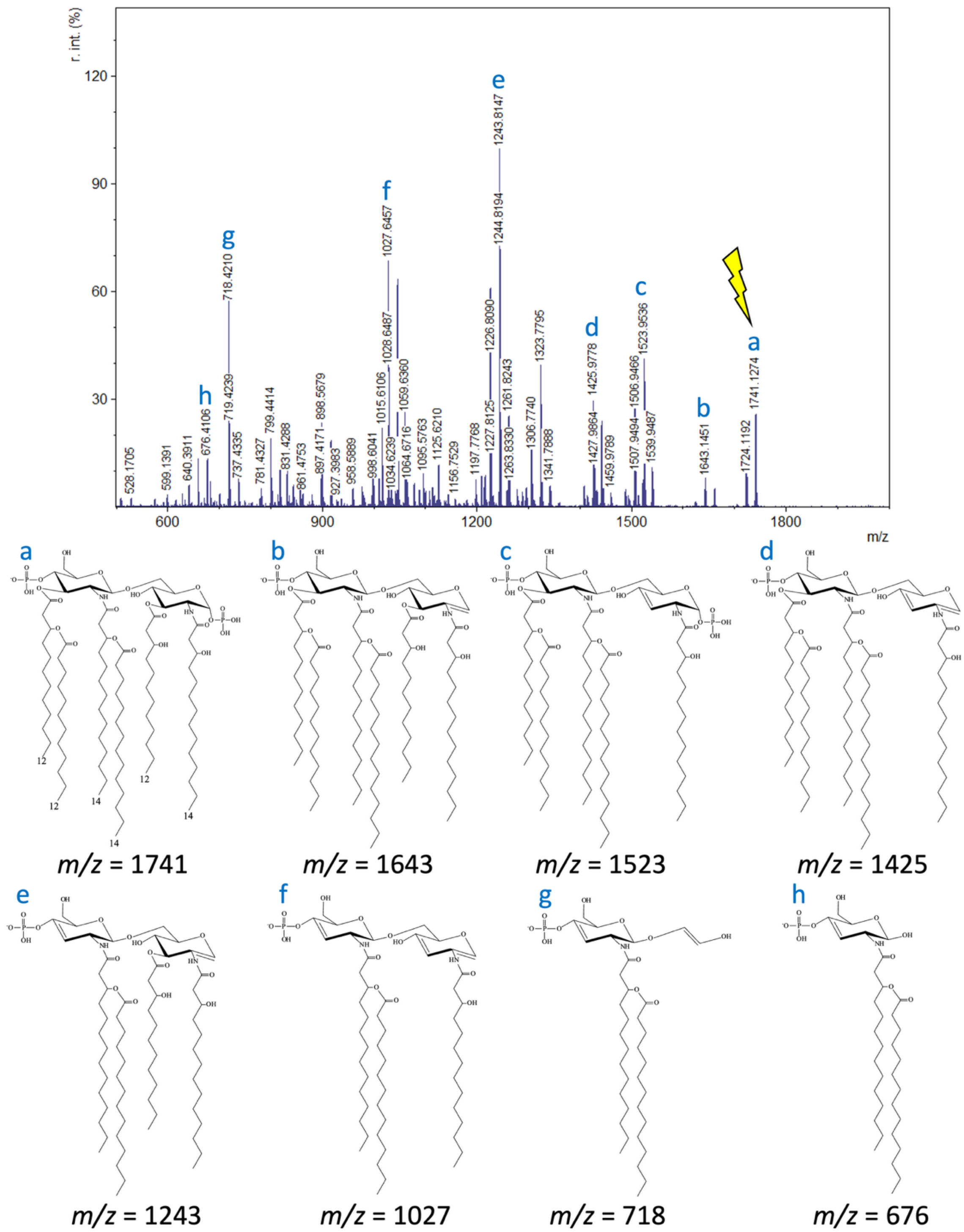

Figure S1: Tandem MS of the Lipid A structure without pEtN-modification

The ion at *m/z* = 1741 was selected as the precursor ion for fragmentation (denoted with the lightning bolt), and the subsequent fragment ions observed are denoted in letters above each spectral peak with corresponding structures shown below the spectra.

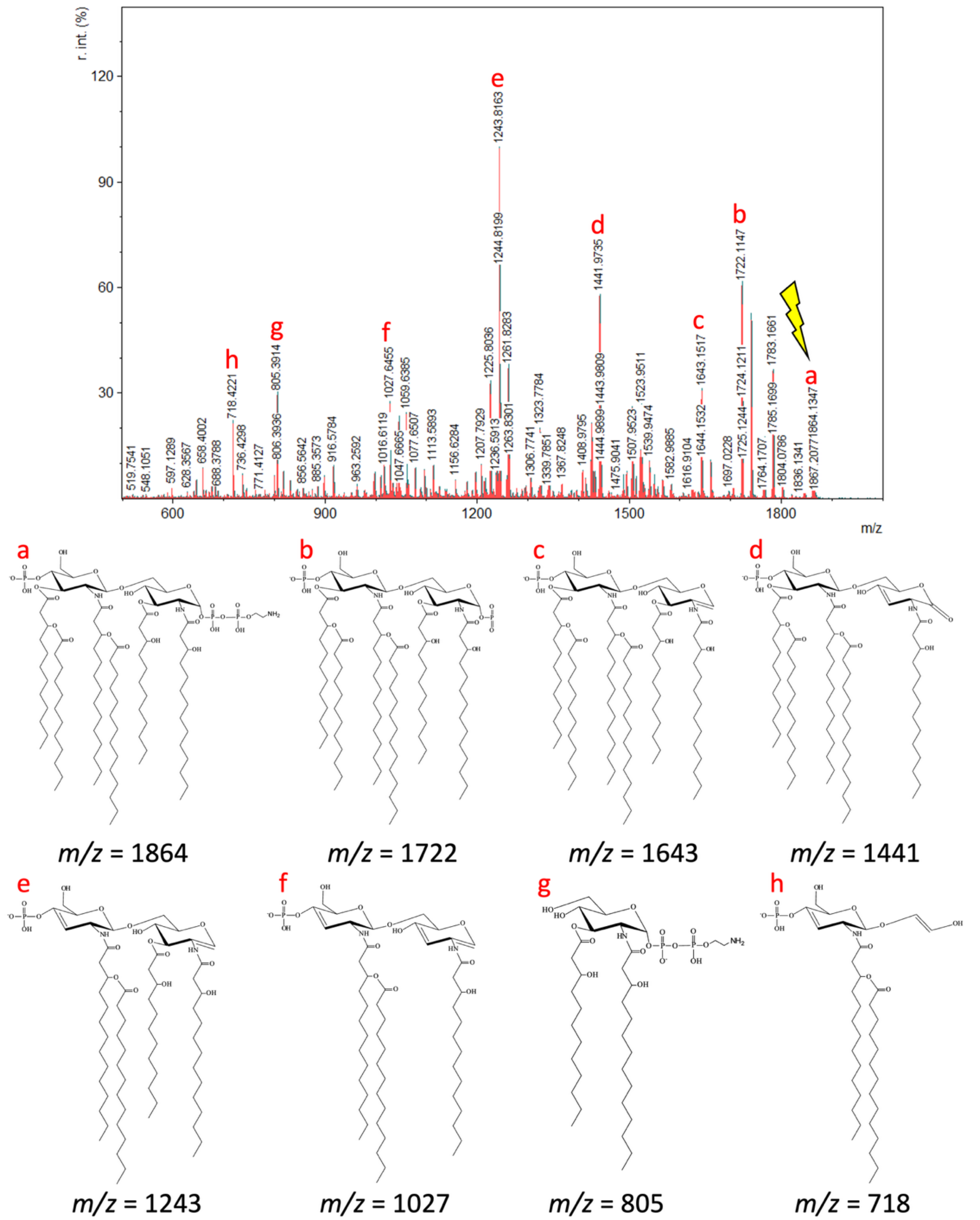

Figure S2: Tandem MS of the Lipid A structure with pEtN-modification

The ion at *m/z* = 1864 was selected as the precursor ion for fragmentation (denoted with the lightning bolt), and the subsequent fragment ions observed are denoted in letters above each spectral peak with corresponding structures shown below the spectra.
